# Structural, electronic and thermoelectric properties of GeC and MXO (M = Ti, Zr and X = S, Se) monolayers and their van der Waals heterostructures

**DOI:** 10.1039/d2ra07797c

**Published:** 2023-03-24

**Authors:** Khadeeja Bashir, M. Bilal, B. Amin, Yuanping Chen, M. Idrees

**Affiliations:** a Department of Physics, Abbottabad University of Science and Technology Havelian Abbottabad 22010 Pakistan binukhn@gmail.com; b School of Physics and Electronic Engineering, Jiangsu University Zhenjiang 212013 Jiangsu China mohadidreess@gmail.com

## Abstract

Vertical stacking of two-dimensional materials into layered van der Waals heterostructures is considered favourable for nanoelectronics and thermoelectric applications. In this work, we investigate the structural, electronic and thermoelectric properties of GeC and Janus monolayers MXO (M = Ti, Zr; X = S, Se) and their van der Waals (vdW) heterostructures using first-principles calculations. The values of binding energies, interlayer distances and thermal stability confirm the stability of these vdW heterostructures. The calculated band structure shows that GeC monolayer have a direct band gap while MXO (M = Ti, Zr; X = S, Se) and their van der Waals heterostructures show indirect band nature. Partial density of states confirms the type-II band alignment of GeC–MXY vdW heterostructures. Our results shows that ZrSeO (GeC) monolayers and GeC–ZrSO vdW heterostructures have higher power factor, making them promising for thermoelectric device applications.

## Abstract 2

Calculated Seebeck coefficient (a) and (b) electrical conductivity (c) and (d) and power factor (e) and (f) of GeC–TiSO, GeC–TiSeO, GeC–ZrSO and GeC–ZrSeO vdW heterostructures for 300 K and 800 K, respectively.

## Graphical abstract

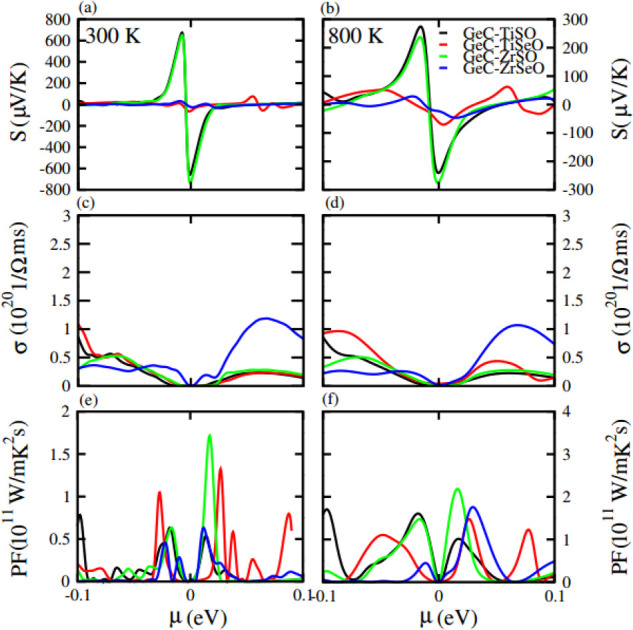

## Introduction

Theoretical and experimental studies of 2D materials have substantially grown during the last decade. In 2004, the breakthrough of successful isolation of single layer graphene was made by mechanical exfoliation^1^ and the discovery of novel, efficient, low-cost and durable materials has significantly increased in the recent era. 2D materials attracted researchers due to their excellent carrier mobility, ultralow weight, high conductivity, high mechanical strength and long spin diffusion length.^2–5^ Transition metal dichalcogenide (TMDC) monolayers with general formula MX_2_, where the transition atoms are placed between two similar chalcogen atoms, show tremendous interest due to their wide range of applications. MX_2_ monolayers are found in rhombohedral or hexagonal phase with M atoms in either trigonal prismatic (2H or 3R) or octahedral (1T) coordination,^6,7^ where the numbers show the layers of X–M–X monolayer and R and T (H) shows rhombohedral phases and trigonal (hexagonal).^8^ Large exciton binding energies,^9^ a suitable energy spectrum from the visible to near infrared region^10^ and spin valley splitting^11^ make MX_2_ monolayers interesting for FETs^12^ and memory devices.^13^ Graphene as the first ever 2D material discovered in 2004 opened a new window in the arena of advanced technology.^1^ The incredible thinness of graphene with an exceptional semiconducting direct band gap (1.0–2.0 eV) nature, high carrier mobility (>200 cm^2^ V^−1^ s^−1^), and high ambient stability make MX_2_ (M = Mo, W; X = S, Se, Te) monolayers ideal candidates for optoelectronic devices.^17^ Recently, selenization in MoS_2_,^14–16^ sulfurization in MoSe_2_ and sulphurization in MoSe_2_ (ref. 18) through chemical vapor deposition (CVD) have been confirmed successfully as Janus MXY (M = Mo, W; X, Y = S, Se, Te) monolayers. Density functional theory (DFT) calculations for electronic structures and Raman vibration modes of SMoSe monolayer are also found to correlate well with experiment.^18^ Recently MXO (M = Ti, Zr, Hf; Y = S, Se) monolayers are investigated and also confirm their stability, which shows promising application in electronic, sensors and photocatalysis.^19^

Besides TMD and JTMD the graphene like hexagonal GeC monolayer of group IV elements, which is confirmed by CVD method^20^ and laser ablation^21,22^ also attract considerable attention due to direct band nature, which makes GeC good candidate for photovoltaics and optoelectronics application.^23–25^ GeC monolayer with honeycomb structure is an indirect bandgap (3.493 eV) semiconductor.^26^ Radio frequency reactive sputtering and activated reactive evaporation techniques were used to make GeC thin films.^27^ Also infrared (IR) spectra revealed GeC vibration modes in narrow sheets. In GeC monolayers, both the CBM and VBM lies at the same point of BZ, shows direct band nature.^27^ GeC monolayer is sensitive to light and can be used for water splitting in the existence of negative electric field with help of ultraviolet light.^28^

The energy resources are aggravated by the passive resistance due to increasing demands of the energy requirements. Therefore, the world is interested in the renewable energy (also called green power) in order to explore the more reliable energy reservoir for accomplishing the energy demands and reducing environmental problems.^29^ The more significant recycling of the waste heat is achieved from thermal power plants and automobiles. It is possible to recover a tremendous amount of heat *via* thermo-electric devices that are preferable for their supplemental advantage of converting it into the electrical energy. Consequently, the thermoelectric performance of 2D materials is gaining increasing interest for energy harvesting purposes.^30^

Similar to the control of dimensionality, external electric field,^31–33^ strain engineering,^34–36^ and vertical stacking *via* van der Waals (vdW) interactions^37–39^ are also effective approaches for manipulation of the electronic properties of materials. In the form of vdW heterostructure, layers stacking is a practical tool to design viable electronic products, like tunneling transistors,^40,41^ flexible optoelectronic devices^42,43^ and bipolar transistors.^44^ Type-II band alignment obtained by confining of VBM and CBM to two different layers of vdW heterostructures is capable to modulate interlayer transition energy and responsible for charge separation,^45–48^ hence intensively used in designing advanced optoelectronic devices.^49,50^

Motivated from the unusual physical and chemical properties from MXO and GeC monolayers, here in this work we calculated the structural, electronic, optical and thermoelectric properties of these monolayers and also their vdW of MXO and GeC monolayers, using density functional theory calculations. Our result confirms that MXO–GeC vdW heterostructures have type-II band alignment which is promising candidate for solar cell application. Furthermore, electrostatic potentials and thermoelectric properties is also calculated.

## Computational details

We used density functional theory (DFT) which is implemented in Vienna *Ab initio* Simulation Package (VASP).^51,52^ In the first Brillouin zone, a *Γ*-point centered of 12 × 12 × 1 Monkhorst–Pack *k*-point grid is used with plane wave cutoff energy of 450 eV. To avoid the interaction between adjacent layer of atoms, a vacuum layer with thickness of 25 Å is considered. Forces and energies were converged to 10^−3^ eV Å^−1^ and 10^6^ eV respectively. To describe possible vdW interaction a DFT-D2 method is used. For correlation and exchange functional we have consider the generalized gradient approximation (GGA),^53,54^ while for accurate band structure we have used hybrid functional (HSE06).^55^ Boltzmann semi-classical theory^56^ is used to calculate electrical transport properties like see beck coefficient (*S*), electrical conductivities (*σ*), thermal conductivities (*K*) and power factor (PF) by using BoltzTrap code. All these parameters can be expressed by following equations:
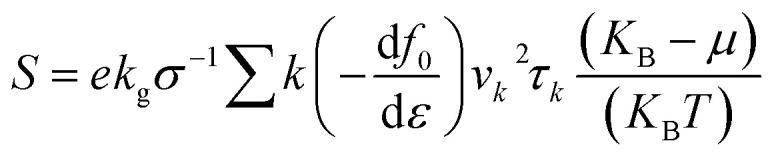

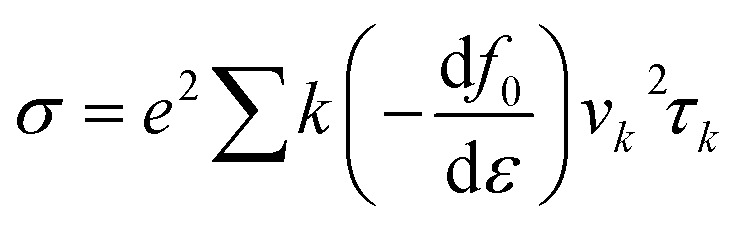
Power factor (PF) = *S*^2^*σ*Here, *e* is the charge of carrier, *ε* is the energy, *K*_B_ is the Boltzmann's constant, *τ*_*k*_ is the relaxation time *f*_0_ is the Fermi distribution function, *μ* denotes the chemical potential and *v*_*k*_ is the group velocity.

## Results and discussion

**Fig. 1:**
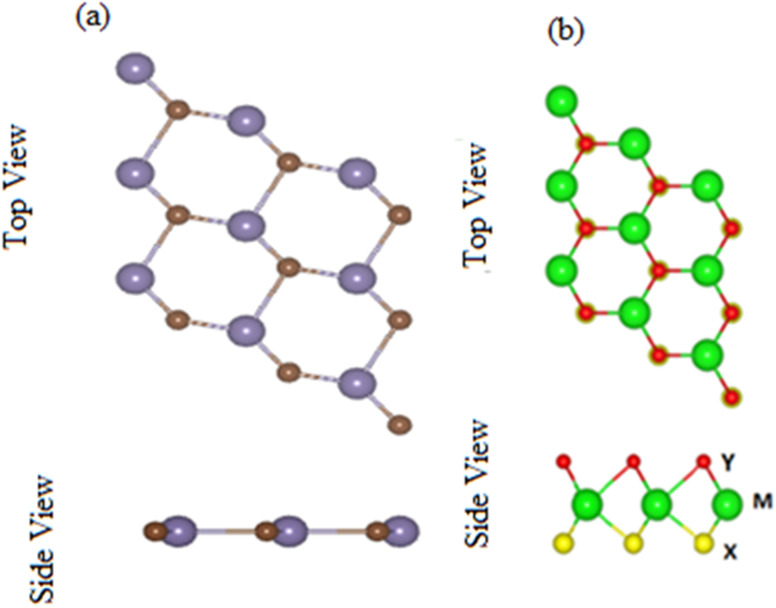
Top and side view of (a) GeC and (b) MXO monolayer, respectively, where different atoms names are mentioned in figure.

Contrary to MX_2_ monolayers, in Janus MXO monolayer, M (transition metal atom) is sandwiched between X and Y (two different chalcogen atoms).^19^ Optimized lattice parameters and bond length of MXO (M = Ti, Zr; X = S, Se) monolayers presented in Table 1 are about the average value of the corresponding MX_2_ monolayers and are in agreement with previously available data^19^ findings. MXO show honeycomb structure just like graphene where the chalcogen and transition atom are bounded by covalent bond as shown in Fig. 1. The top and side view of GeC monolayer is presented in Fig. 1 which is dynamically stable planer structure and the relaxed parameters like bandgaps, bond lengths, bond angles and lattice constants of TiSO, TiSeO, ZrSO and ZrSeO monolayers are listed in Table 1, agreement with ref. 19 and 57.

**Table 1:** Lattice parameters, bond length, electronic band gaps, bond angles of MXY monolayers

Monolayers	*a* (Å)	X–Y (Å)		*E*_g_ (eV)
GeC	3.23	Ge–C	1.0901	2.95
TiSO	3.11	Ti–S	2.4516	1.449
		Ti–O	2.0279	
TiSeO	3.15	Ti–Se	4.2336	0.926
		Ti–O	2.0831	
ZrSO	3.33	Zr–S	4.3547	1.93
		Zr–O	3.7963	
ZrSeO	3.41	Zr–Se	2.7853	1.05
		Zr–O	4.1808	

**Fig. 2:**
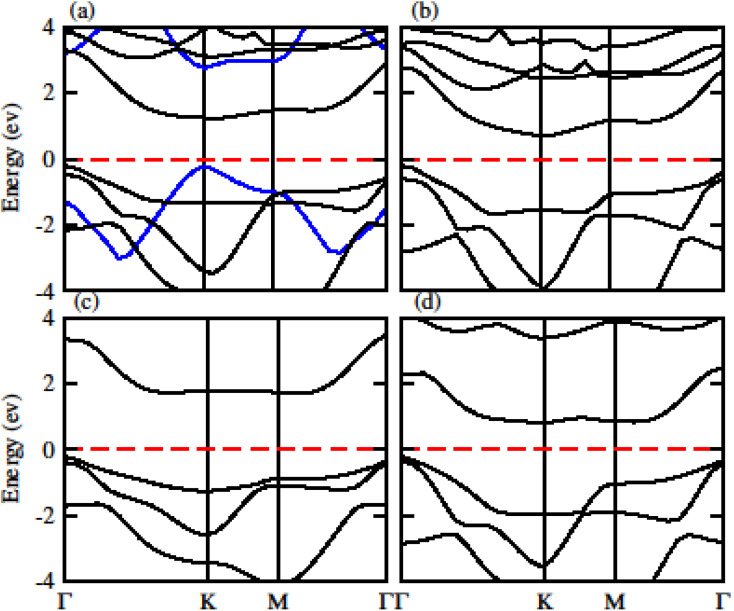
Calculated electronic band structure of (a) GeC (blue lines), TiSO, (b) TiSeO (c) ZrSO and (d) ZrSeO monolayer, respectively.

**Fig. 3:**
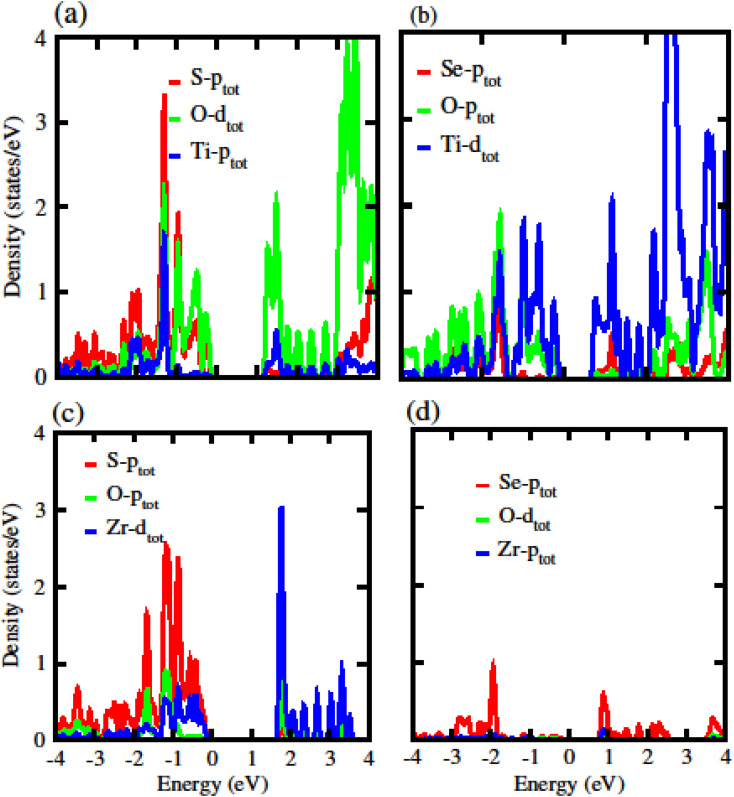
Partial density of states (PDOS) of (a) TiSO (b) TiSeO (c) ZrSO (d) ZrSeO monolayers, respectively.

The calculated electronic band structure of GeC, TiSO, TiSeO and ZrSeO monolayers are presented in Fig. 2. The calculated band structure of MXO monolayers show indirect band nature with VBM lies at *Γ*-point while CBM located at *K*-point of first BZ (see Fig. 2). Moreover, the electronic band structure of GeC monolayer is evaluated to be 2.95 eV which shows direct band nature with CBM (VBM) located at point *Γ*(*K*) of first BZ. The calculated band gap values of GeC and MXO monolayers are presented in Table 1. For further verification of band structures and different state contributions, we have calculated the partial density of states (PDOS) of GeC, TiSO, TiSeO, ZrSO and ZrSeO monolayer which are presented in Fig. 3, which shows the contribution of materials in valence and conduction bands. In case of TiSO (TiSeO) (Fig. 3(a)) both the CBM and VBM lie from the d state of Ti atom, where small contribution of S(Se) and O atom are also present. In ZrSeO monolayer (Fig. 3(d)) the CBM and VBM is due to the p state of Se atom.

**Fig. 4:**
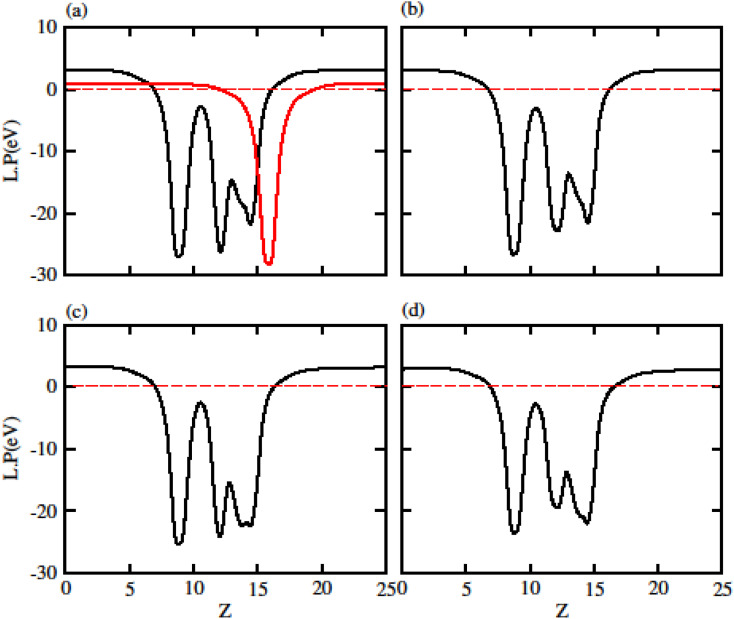
Calculated electrostatic potential of (a) TiSO (b) TiSeO (c) ZrSO and (d) ZrSeO monolayers, respectively.

Due to polarization in Janus monolayers the work function for MXO is also presented in Fig. 4. The difference in work function for that system is due to the two different atoms attached in MXO monolayer (S and Se). In MXO system the internal polarization arises an internal electric field which is due to the M and X surface. We have further calculated the work function (*ϕ*) which is given in Table 2 for GeC, TiSO, TiSeO, ZrSO and ZrSeO monolayer. The work function shows that it is decreases from TiSO to ZrSeO.

**Table 2:** Work function of GeC, TiSO, TiSeO, ZrSO and ZrSeO monolayers

Monolayers	Work function (eV)
GeC	0.89
TiSO	0.2247
TiSeO	1.3199
ZrSO	1.3201
ZrSeO	1.1845

**Fig. 5:**
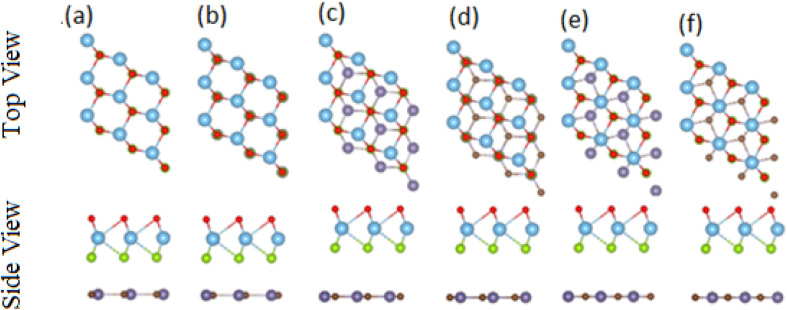
Top and side view of different stacking configuration of GeC–MXO vdW heterostructures.

**Fig. 6:**
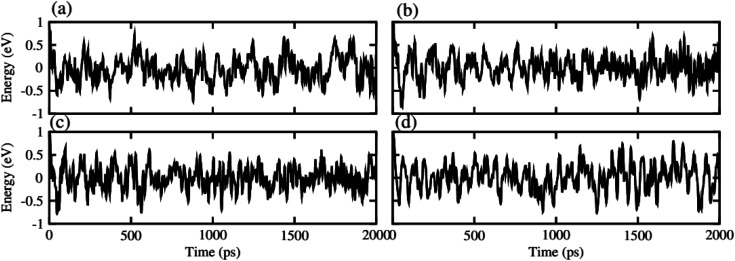
Thermal stability of (a) GeC–TiSO, (b) GeC–TiSeO, (c) GeC–ZrSO and (d) GeC–ZrSeO vdW heterostructure, respectively.

After careful studies of these monolayer, we have calculated the vdW heterostructure of these systems. Due to the same hexagonal lattice symmetry and small and experimentally achievable lattice mismatch for MXO–GeC vdW heterostructures show possible experimental fabrication for future applications.^58^ As the electronic structure are sensitive different stacking (position of atoms in the layer), we have plotted six different possible configurations of GeC–TiSO, GeC–TiSeO, GeC–ZrSO and GeC–ZrSeO vdW heterostructure which is given in Fig. 5. In stacking (a) the Ti(Zr) atom is placed on the top of Ge atom while S and O is placed on the C atom. In stacking (b) the Ti atom is placed on the top of C atom, while O and Se atom is placed on the top of Ge atom. In stacking (c) O and S is placed on the top of C atom, in stacking (d) O and S is placed on the Ge atom while C is placed in the hexagonal site. Stacking (f) is just the reciprocal of stacking (e). To find out the most stable configuration in these stacking we have calculated the minimum energy, binding energy and interlayer distance which is presented in Table 3. One can find out that stacking (a) has more negative binding energy and smaller interlayer distance hence, shows most favourable stacking configuration and therefore, we will proceed with this configuration for further calculations. The calculated values of lattice constant and bond length are given in Table 3 for GeC–MXO vdW heterostructures. For further verification we have calculated the thermal stability of most stable stacking (a) and check the structure distortion of GeC–MXO vdW heterostructures which is displayed in Fig. 6 using AIMD calculation.^59,60^ One can easily confirm that GeC–MXO vdW heterostructures retain their geometry without structure distortion and also shows very small variation in total energies, which confirm the thermal stability of these systems at 300 K (room temperature). So, for further calculation we will consider stacking configuration (a).

**Table 3:** The calculated values of lattice constants, bond lengths, band gaps values for PBE and HSE06

Heterostructure	*a* (Å)	X–Y (Å)	*E*_g_ (PBE) (eV)	*E*_g_ (HSE) (eV)
GeC–TiSO	3.19	Ge–C = 3.1022	0.0215	0.046
		Ti–S = 4.8778		
		Ti–O = 4.8862		
GeC–TiSeO	3.23	Ge–C = 1.6726	0.035	0.245
		Ti–Se = 2.0798		
		Ti–O = 2.1515		
GeC–ZrSO	3.28	Ge–C = 1.7035	0.033	0.143
		Zr–S = 2.6210		
		Zr–O = 2.6254		
GeC–ZrSeO	3.32	Ge–C = 1.7913	0.036	0.2
		Zr–Se = 2.7144		
		Zr–O = 2.7785		

**Fig. 7:**
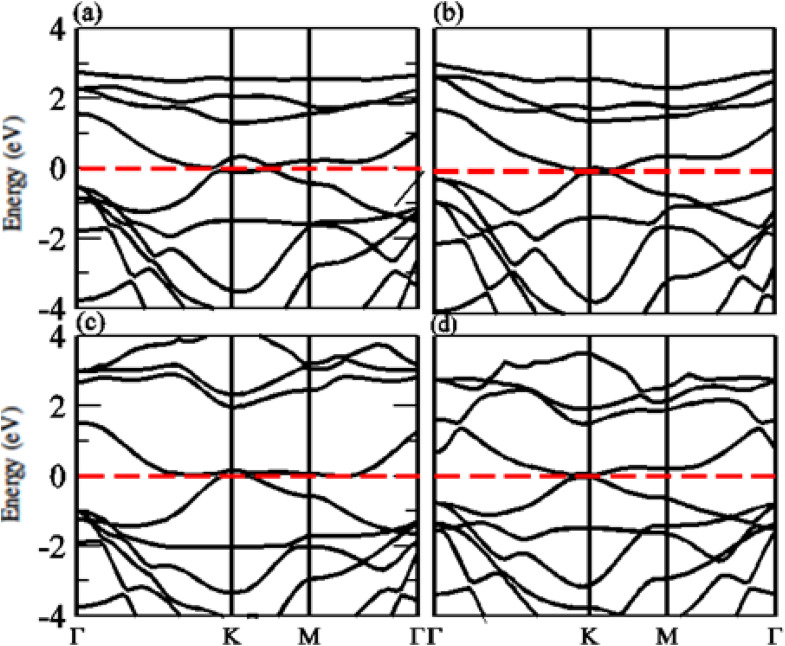
Calculated electronic band structure of (a) GeC–TiSO, (b) GeC–TiSeO, (c) GeC–ZrSO and (d) GeC–ZrSeO vdW heterostructure, respectively using PBE method.

**Fig. 8:**
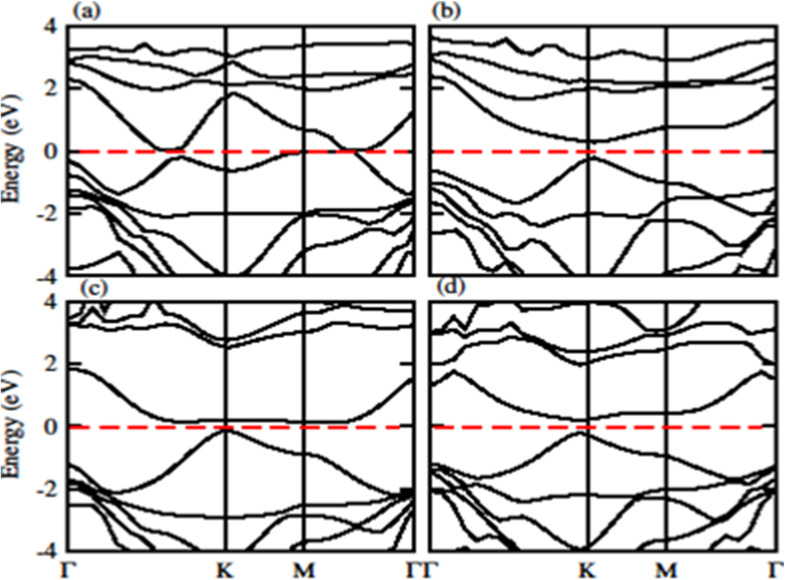
Calculated electronic band structure of (a) GeC–TiSO, (b) GeC–TiSeO, (c) GeC–ZrSO and (d) GeC–ZrSeO vdW heterostructure, respectively using HSE06 method.

The calculated band structure of GeC–MXY vdW heterostructure using PBE and HSE methods are given in Fig. 7 and 8, respectively. Band structures show that all these vdW heterostructures are semiconductor with direct bandgap nature, where VBM and CBM lie at *Γ*–*K* points of first BZ. The direct band nature of these materials shows good response for solar cell and energy harvesting application. The calculated values for PBE and HSE06 are given in Table 3, which illustrates that HSE06 values are greater than that of PBE values.

**Fig. 9:**
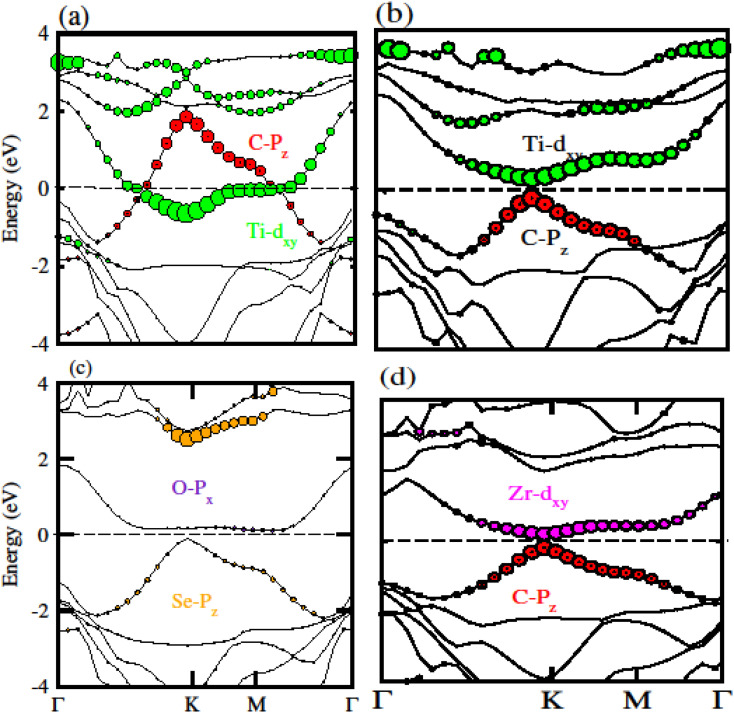
Calculated weighted band structure of (a) GeC–TiSO, (b) GeC–TiSeO, (c) GeC–ZrSO and (d) GeC–ZrSeO vdW heterostructure, respectively.

To confirm the system for type-I and type-II band alignment we have calculated the weighted band structure of these systems which is presented in Fig. 9. In case of GeC–TiSO the CBM is due to the Ti–d_*xy*_ state of TiSO monolayer while VBM is due to the C–p_*z*_ state of GeC monolayer. Similarly, in case of GeC–TiSeO the VBM is due to the C–p_*z*_ state of GeC monolayer while the CBM is due to the Ti–d_*xy*_ state of TiSeO monolayer. Now if we look to GeC–ZrSO vdW heterostructure where both VBM or CBM lie from ZrSO monolayer (VBM is due to Se–p_*z*_ and CBM is O–p_*x*_ state). In case of GeC–ZrSeO VBM is due to C–p_*z*_ state while CBM is Zr–d_*xy*_. Hence GeC–TiSO, GeC–TiSeO and GeC–ZrSeO show type-II band alignment while GeC–ZrSO has type-I band nature. In type-I band nature VBM and CBM lie from same monolayer show good response for solar energy application. While in type-II band nature VBM is from two different monolayers hence shows good response for laser and energy harvesting application.

Furthermore, we have calculated the thermoelectric properties of GeC, MXO monolayers and their vdW heterostructures. In which Seebeck effect (coefficient) is the fundamental approach for thermoelectric properties. We have calculated thermoelectric properties against chemical potential (*μ*) which is the carrier concentration of any material. *μ* have positive and negative values depends on different side of Fermi level (positive is for n-type and negative is for p-type doping). *μ* indicating doping level of a compound; for n-type doping, *μ* has positive value and responsible for shifting up the Fermi level while for p-type doping, *μ* has negative value and shifts downward the Fermi level. Here we used BoltzTrap code, for calculated the transport parameters such as Seebeck coefficient, electrical conductivity and power factor of GeC and MXO monolayers and their vdW heterostructures. Here in our calculation the phonon contribution is not considered in thermoelectric properties, because we only calculate here the Seebeck coefficient, thermal and electric conductivity.

**Fig. 10:**
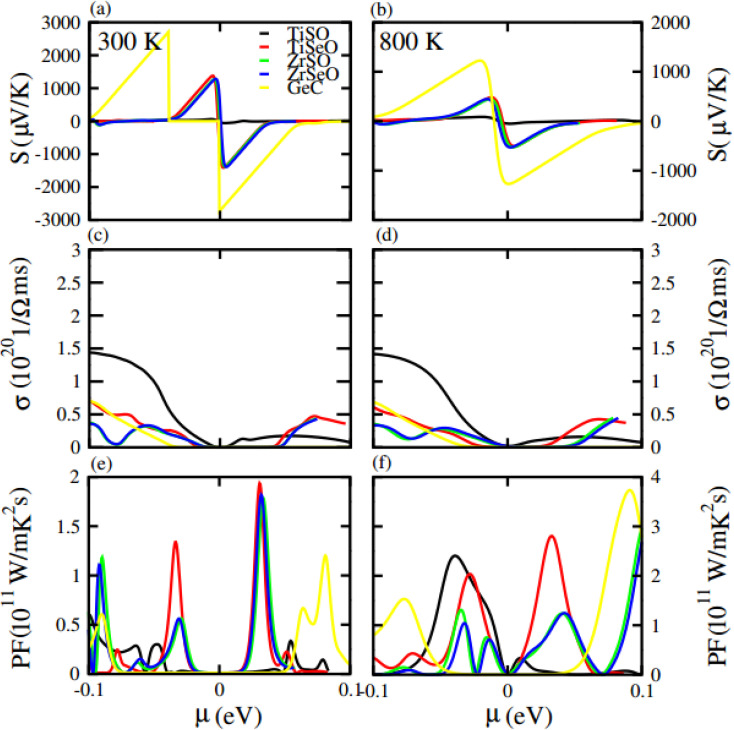
Calculated Seebeck coefficient (a) and (b) electrical conductivity (c) and (d) and power factor (e) and (f) of GeC, TiSO, TiSeO, ZrSO and ZrSeO monolayer for 300 K and 800 K, respectively.

**Fig. 11:**
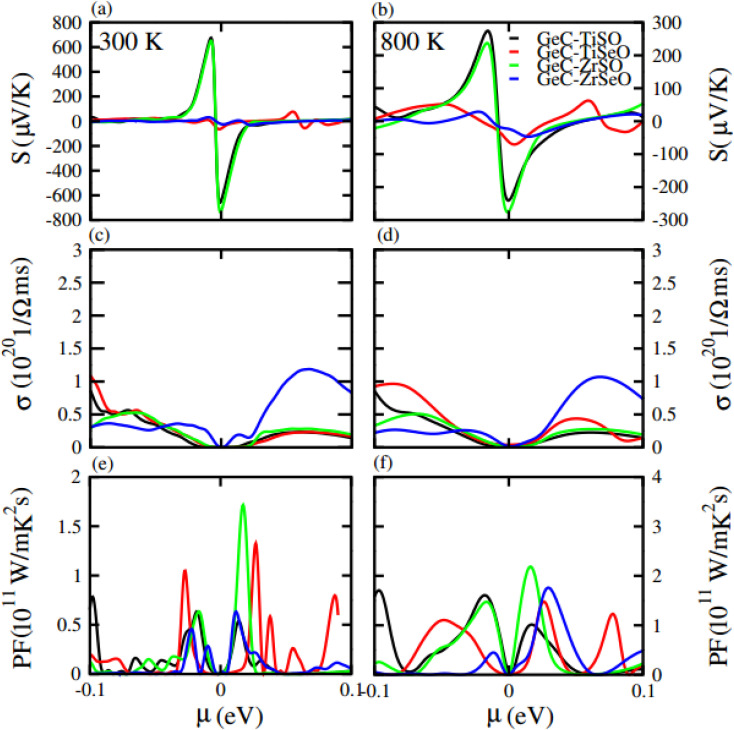
Calculated Seebeck coefficient (a) and (b) electrical conductivity (c) and (d) and power factor (e) and (f) of GeC–TiSO, GeC–TiSeO, GeC–ZrSO and GeC–ZrSeO vdW heterostructures for 300 K and 800 K, respectively.

Seebeck coefficient in term of chemical potential (*μ*) at 300 K and 800 K temperature is plotted for GeC, TiSO, TiSeO, ZrSO and ZrSeO monolayer in Fig. 10 and for their vdW heterostructure is plotted in Fig. 11, one can easily figure out that the Seebeck coefficient for GeC, TiSO, TiSeO, ZrSO and ZrSeO monolayer and their vdW heterostructures gives higher values in p-type region than n-type region which is decreases by increases the temperature from 300 K to 800 K. The calculated peaks values of Seebeck coefficient for 300 K and 800 K for GeC, TiSO, TiSeO, ZrSO and ZrSeO monolayer and also their vdW heterostructures is given in Tables 4 and 5. One can observed that in monolayers GeC have higher values while in heterostructures GeC–ZrSO vdW heterostructures have higher values for Seebeck coefficient. From previous study it has been reported that Seebeck vales which is higher than that of 200 μV K^−1^ will be good material for thermoelectric devices,^41^ hence we can predict that all monolayers and GeC–ZrSO (GeC–TiSO) vdW heterostructures have larger Seebeck which is best materials for thermoelectric device applications. Similar results are also reported in ref. 61 and 62.

**Table 4:** Seebeck coefficient, electrical conductivity and power factor of TiSO, TiSeO, ZrSO, ZrSeO and GeC monolayers for n and p-type regions

Monolayers	TiSO	TiSeO	ZrSO	ZrSeO	GeC
**300 K**	**P**				
*S* (μV K^−1^)	50	1400	1400	1380	2740
*σ* (1/Ω ms)	1.43 × 10^20^	0.9 × 10^20^	0.4 × 10^20^	0.38 × 10^20^	0.8 × 10^20^
PF (W mK^−2^ s^−1^)	0.5 × 10^11^	0.35 × 10^11^	1.23 × 10^11^	1.18 × 10^11^	0.6 × 10^11^

**800 K**	**P**				
*S* (μV K^−1^)	50	500	450	450	1280
*σ* (1/Ω ms)	1.4 × 10^20^	0.6 × 10^20^	0.32 × 10^20^	0.35 × 10^20^	0.7 × 10^20^
PF (W mK^−2^ s^−1^)	2.4 × 10^11^	2.1 × 10^11^	1.3 × 10^11^	1 × 10^11^	1.57 × 10^11^

**300 K**	**N**				
*S* (μV K^−1^)	50	1400	1350	1350	2740
*σ* (1/Ω ms)	0.2 × 10^20^	0.5 × 10^20^	0.42 × 10^20^	0.41 × 10^20^	0.05 × 10^20^
PF (W mK^−2^ s^−1^)	0.34 × 10^11^	0.21 × 10^11^	1.8 × 10^11^	1.83 × 10^11^	1.21 × 10^11^

**800 K**	**N**				
*S* (μV K^−1^)	80	580	560	560	1250
*σ* (1/Ω ms)	0.28 × 10^20^	0.4 × 10^20^	0.39 × 10^20^	0.38 × 10^20^	0.065 × 10^20^
PF (W mK^−2^ s^−1^)	0.35 × 10^11^	2.8 × 10^11^	2.7 × 10^11^	2.6 × 10^11^	3.75 × 10^11^

**Table 5:** Seebeck coefficient, electrical conductivity and power factor of GeC–TiSO, GeC–TiSeO, GeC–ZrSO and GeC–ZrSeO vdW heterostructures for n and p-type regions

vdW heterostructures	GeC–TiSO	GeC–TiSeO	GeC–ZrSO	GeC–ZrSeO
**300 K**	**P**			
*S* (μV K^−1^)	670	20	650	30
*σ* (1/Ω ms)	0.8 × 10^20^	1.08 × 10^20^	0.55 × 10^20^	0.36 × 10^20^
PF (W mK^−2^ s^−1^)	0.75 × 10^11^	1.02 × 10^11^	0.65 × 10^11^	0.45 × 10^11^

**800 K**	**P**			
*S* (μV K^−1^)	280	50	235	35
*σ* (1/Ω ms)	0.83 × 10^20^	1 × 10^20^	0.56 × 10^20^	0.26 × 10^20^
PF (W mK^−2^ s^−1^)	1.7 × 10^11^	1.1 × 10^11^	1.5 × 10^11^	0.5 × 10^11^

**300 K**	**N**			
*S* (μV K^−1^)	670	65	700	25
*σ* (1/Ω ms)	0.234 × 10^20^	0.22 × 10^20^	0.27 × 10^20^	1.2 × 10^20^
PF (W mK^−2^ s^−1^)	0.52 × 10^11^	1.4 × 10^11^	1.7 × 10^11^	0.6 × 10^11^

**800 K**	**N**			
*S* (μV K^−1^)	240	75	280	50
*σ* (1/Ω ms)	0.25 × 10^20^	0.35 × 10^20^	0.3 × 10^20^	1.04 × 10^20^
PF (W mK^−2^ s^−1^)	1 × 10^11^	1.45 × 10^11^	1.8 × 10^11^	1.75 × 10^11^

Electrical conductivity (*σ*) of a material which is due to the holes and electrons in semiconductors is calculated against *μ* for GeC, TiSO, TiSeO, ZrSO and ZrSeO monolayer and also their vdW heterostructures (see Fig. 10 and 11). For good thermoelectric materials we need high *σ*. The calculated values for *σ* for both n and p-type is given in Table 4 for 300 K and 800 K. From Fig. 10 and 11 and also from Tables 4 and 5, one can see that TiSO in monolayers and GeC–TiSeO vdW heterostructure have higher values from other materials at 300 K.

To summarize the thermoelectric efficiency of GeC, TiSO, TiSeO, ZrSO and ZrSeO monolayer and also their vdW heterostructures we have calculated the power factor (PF) by using PF = *S*^2^*σ*, where *S* (*σ*) represents Seebeck coefficient (electrical conductivity) of the material. Here we calculated the PF of GeC, TiSO, TiSeO, ZrSO and ZrSeO monolayer and also their vdW heterostructures against *μ* and plotted in Fig. 10 and 11 and given in Tables 4 and 5. One can easily figure out that in monolayers ZrSeO (GeC) have higher value in n-type region while in heterostructures GeC–ZrSO vdW heterostructure have higher value in n-type region for 300 K (800 K), making it promising for thermoelectric device applications.

## Conclusions

To conclude, the structural, electronic and optical properties of two-dimensional Janus transition metal oxides MXY (M = Ti, Zr; X = S and Se and Y = O) are performed within the framework of density functional theory. Moreover, a direct band gap is observed in GeC monolayer while TiSO, TiSeO, ZrSO and ZrSeO show an indirect band gap. The optimized lattice constants for TiSO, TiSeO, ZrSO and ZrSeO are 3.11 Å, 3.15 Å, 3.33 Å and 3.41 Å, respectively. Electronic band structures revealed semiconducting manner for GeC and Janus monolayers, and PDOS indicate that the states at the Fermi level are due to the semiconducting monolayers. By using DFT calculations, the electronic and structural properties of four van der Waals heterostructures contacts GeC–MXO vdW heterostructures are also determined. All the heterostructures are found to be semiconductors with type-II band alignments. Power factor shows that ZrSeO (GeC) monolayers and GeC–ZrSO vdW heterostructure have higher power factor, which makes it promising for thermoelectric device applications.

## Conflicts of interest

There are no conflicts to declare.

## Supplementary Material
